# The Effect of Multispecies Probiotic Supplementation on Iron Status in Rats

**DOI:** 10.1007/s12011-019-1658-1

**Published:** 2019-02-12

**Authors:** Katarzyna Skrypnik, Paweł Bogdański, Marcin Schmidt, Joanna Suliburska

**Affiliations:** 1 Institute of Human Nutrition and Dietetics, Poznan University of Life Sciences, Wojska Polskiego St. 31, 60-624 Poznan, Poland; 2 Department of Treatment of Obesity, Metabolic Disorders and Clinical Dietetics, Poznan University of Medical Sciences, Szamarzewskiego St. 82/84, 60-569 Poznan, Poland; 3 Department of Food Biotechnology and Microbiology, Poznan University of Life Sciences, Wojska Polskiego St. 48, Poznan, 60-627 Poland

**Keywords:** Probiotic, Iron metabolism, Zinc, Copper, Total iron binding capacity

## Abstract

A range of interactions between gut microbiota and iron (Fe) metabolism is described. Oral probiotics ameliorate host’s iron status. However, this has been proven for single-strain probiotic supplements. Dose-dependence of beneficial probiotic supplementation effect on iron turnover remains unexplored. Our study aimed to investigate the effects of oral multispecies probiotic supplementation in two doses on iron status in rats. Thirty rats were randomized into three groups receiving multispecies probiotic supplement at a daily dose of 2.5 × 10^9^ CFU (PA group, *n* = 10) and 1 × 10^10^ CFU (PB group, *n* = 10) or placebo (KK group, *n* = 10). After 6 weeks, rats were sacrificed for analysis, blood samples, and organs (the liver, heart, kidneys, spleen, pancreas, femur, testicles, duodenum, and hair) were collected. The total fecal bacteria content was higher in the PB group vs. PA group. Unsaturated iron-binding capacity was higher in the PB group vs. KK group. Serum Fe was lower in both PA and PB vs. KK group. Iron content in the liver was higher in the PB group vs. KK group; in the pancreas, this was higher in the PB group vs. the KK and PA group, and in the duodenum, it was higher in both supplemented groups vs. the KK group. A range of alterations in zinc and copper status and correlations between analyzed parameters were found. Oral multispecies probiotic supplementation exerts dose-independent and beneficial effect on iron bioavailability and duodenal iron absorption in the rat model, induces a dose-independent iron shift from serum and intensifies dose-dependent pancreatic and liver iron uptake.

## Introduction

The human microbiota can have a total mass of as much as 2 kg (comparable to that of a human brain) and has been called a “newly discovered organ” [1–4]. A wide range of interactions between gut microbiota and iron (Fe) status have been extensively documented [5–11]. Iron is an important component of both hemoglobin, which is responsible for blood oxygen transport, and of myoglobin. Iron is also a component of enzymes such as cytochromes, catalase, and peroxidase [12]. Iron deficiency leads to anemia [13], and iron excess may lead to increased oxidative stress and insulin resistance [14–16]. Iron overload is a risk factor for renal dysfunction, hypogonadism [17, 18], and diabetes [19]. In rats, high iron intake leads to increased gonad mass [20]. Iron interacts with copper and zinc [21], which are important components of a range of enzymes [22, 23]; disturbances in the metabolism of these three minerals frequently coexist [21]. Gut microbiota increases the availability of dietary iron to the host by decreasing the amount of iron-binding compounds in the gut [6] and by reducing Fe (III) to Fe (II) [9], which can be absorbed by intestinal cells, unlike Fe (III) [12]. In germ-free rodents, a lack of intestinal bacteria leads to Fe deficits in enterocytes [10]. On the contrary, gut *Bifidobacterium* can limit intestinal Fe content in order to preserve pathogenic bacteria development, even under conditions of iron deficiency [11]. Fe is also essential in intestinal bacteria’s energy acquisition [5]. In rats, Fe deficiency leads to intestinal bacterial translocation [13] and constitutes a major factor in low gut bacteria diversity; Fe supplementation allows only limited possibilities to reverse this state [7]. Correct Fe intestinal levels diminish the colonization ability and virulence of pathogenic microorganisms [8].

Recently, the development of new methods to ameliorate the quality of gut microbiota and its beneficial effect on the host’s health has become the aim of a significant worldwide research effort. Oral probiotic supplementation is the most effective intervention in this range [24]. It has been demonstrated that probiotic supplementation beneficially affects the host’s iron status. Probiotic *Lactobacillus plantarum* increases Fe absorption from iron-supplemented beverages by 50% [25], and also from meals [26]. Germ-free rodents are able to supplement Fe deficit only with a simultaneous supply of the probiotic *Streptococcus thermophilus* [27]. In contrast, unfavorable hepatic iron accumulation in rats can be decreased with oral *Bificobacterium* [28]. It has been demonstrated that iron acquisition is the main mechanism through which probiotic bacteria such as the *Escherichia coli* strain Nissle 1917 limit the intestinal development of pathogenic *Salmonella typhimurium* [26].

According to Food and Agriculture Organization (FAO) and World Health Organization (WHO) definitions, probiotics are live microorganisms that confer a health benefit on the host [29]. This definition emphasizes that the beneficial properties of probiotics are strain-dependent and must not be extrapolated from one strain to different [29]. Thus, the health advantages of probiotic supplementation must be well-documented for each strain separately. For this reason, the vast majority of studies on the effect of probiotics on the host’s health are limited to one probiotic strain and to one dose of probiotic microorganisms [24]. *Lactobacillus, Bifidobacterium, Propionibacterium, Enterococcus,* and the *S. boulardii* yeasts are the most investigated probiotic species [30]*.* There is an evident dearth of actual knowledge on the effect of multispecies probiotic supplementation on the host’s health, especially in terms of Fe metabolism. Also, the question of whether multispecies probiotic supplements exert additive or synergistic effects remains unexplored. To date, only one study has investigated the effects of probiotic supply on total iron binding capacity (TIBC) level, but this was in a single-strain and one-dose mode [26]. Moreover, the effect of different probiotic doses on the host’s iron balance remains insufficiently investigated [24].

The aim of this study was to investigate the effects of 6 weeks of oral multispecies probiotic supplementation in two doses on selected parameters of iron status in the rat model. To the best of our knowledge, this is the first study worldwide to investigate the effect of multistrain probiotics on iron balance in a dose-comparison model.

## Material and Methods

### Animals

Thirty male 10-week-old Wistar rats from the same strain were purchased directly before the experiment from the Department of Toxicology, Poznań Medical University, Poland. The experiment conformed to Polish legal requirements and to the European Communities Council Directive of 24 November 1986, as well as to the National Institutes of Health Guide for the Care and Use of Laboratory Animals (National Institutes of Health Publications No. 80-23, Revised 1978). All study procedures were performed in accordance with the protocols of Poznań University of Life Sciences and were approved by the local bioethics committee for animal studies (approval no. 24/2017). The baseline mean body mass of the animals was 263 ± 22 g [31]. Adaptation to laboratory conditions lasted 5 days prior to the beginning of the experiment. During this period, the animals had unlimited access to a standard AIN-93 M diet [32] (Altromin, Lage, Germany) and deionized water. The animals were housed in controlled and stable conditions at the Laboratory of the Institute of Human Nutrition and Dietetics, Poznań University of Life Sciences. The temperature in the animal room was 21 ± 2 °C, with light/dark cycles lasting 12 h/12 h (light cycle starting at 7:00 am and dark cycle starting 7:00 pm); the relative humidity was 55–65% throughout the adaptation period and the experiment. During the adaptation period and the experiment, the rats were housed in pairs in stainless steel cages coated with metal-free enamel.

### Experimental Design

Thirty rats were randomly assigned to three study groups of ten animals each using a random number generator. The group were a control group (KK), a group receiving low doses of probiotics (PA), and a group receiving high doses of probiotics (PB). The experimental period was 6 weeks. Throughout the experimental period, the animals were fed a standard AIN-93 M maintenance diet (Altromin, Lage, Germany). There were no additives to the diet in the KK group. In the diet of the PA and PB groups, multispecies probiotic was added at a daily dose of 2.5 × 10^9^ CFU and 1 × 10^10^ CFU respectively. The animals in all three groups were allowed to consume diet and drink deionized water ad libitum through the whole experimental period. Each day, a fresh portion of diet and water was supplied to the animals and the remains of diet and water from the previous day were removed. The consumption of diet and water was monitored daily and the rats’ body mass was monitored weekly. At baseline, throughout the whole study, and at completion, there were no differences in rats’ body weight, body weight increase [31], or diet and water consumption between all three groups.

### Probiotic

The Ecologic Barrier probiotic mixture (Winclove Probiotics, Amsterdam, Netherlands) contains nine probiotic bacterial strains (*Bifidobacterium bifidum* W23, *B. lactis* W51, *B. lactis* W52, *Lactobacillus acidophilus* W37, *L. brevis* W63, *L. casei* W56, *L. salivarius* W24, *Lactococcus lactis* W19, and *Lc. lactis* W58) in equal proportions, at a dose of 2.5 × 10^9^ CFU/g. The probiotic took the form of a freeze-dried powder with maize starch and maltodextrins as the carrier matrix [33]. The probiotic was disseminated in a portion of diet in order to prepare a homogeneous mixture each day directly before supplying it to the rats. The probiotic was added to the diet of the PA and PB groups after the accommodation period.

### Blood and Organ Collection

After 6 weeks of the experiment, the rats were weighted and then euthanized by carbon dioxide inhalation. The body length was measured from the top of the nose to the end of the tail. Blood samples were collected by cardiac puncture after a 12-h fasting period to obtain whole blood for morphological analysis and were held in serum-separated tubes to obtain serum. The coagulated blood was left to clot for 30 min at room temperature. Afterwards, the blood was centrifuged at 2000 rpm for 15 min at 4 °C. The supernatant fluid was separated and stored frozen at − 80 °C for analysis. During sectioning, the liver, heart, kidneys, spleen, pancreas, femur, testicles, duodenum, and hair were removed, washed in saline, weighed, and stored at − 20 °C. Hair was collected from the same anatomical area (the interscapular region) of each rat.

### Biochemical and Mineral Measurements

Whole-blood morphological analysis was performed by a commercial laboratory (Synevo, Poznań, Poland). The unsaturated iron-binding capacity (UIBC) was determined using the colorimetric method with ferrozine [34, 35]. The TIBC was calculated as: TIBC = UIBC + serum iron concentration [35]. The Fe serum concentration and the Fe, Zn, and Cu contents of the internal organs (liver, heart, kidneys, spleen, pancreas, femur, testicles, and duodenum, along with the hair) were determined after digestion in 65% (*w*/*w*) spectra pure HNO_3_ (Merck, Kenilworth, NJ, USA) in a Microwave Digestion system (Speedwave Xpert, Berghof, Eningen, Germany). After digestion and dilution with deionized water, the concentrations of Fe, Zn, and Cu in the mineral solutions were determined using flame atomic absorption spectrometry (AAS-3, Carl Zeiss, Jena, Germany). The mineral contents of the internal organs and hair were measured at wavelengths of 248.3 nm for iron, 213.9 nm for zinc, and 324.8 nm for copper. The accuracy of the method was verified using certified reference materials (Bovine liver 1577C, Sigma-Aldrich, Saint Louis, MO, USA) and was 97% for iron, 95% for zinc, and 103% for copper.

### Microbiological Analysis

For the last 3 days of the experiment, before the fasting period, feces were collected from all three study groups for microbiological analysis of the total fecal bacteria content and *Lactobacillus* fecal content. The rat feces were weighted and soaked in saline supplemented with 0.1% Tween 80, then homogenized in a Stomacher device. The suspension of feces was serially diluted and plated in duplicate on MRS (De Man, Rogosa and Sharpe) Agar (BTL, Poland) for *Lactobacillus* spp. enumeration and Columbia Agar with 5% Sheep Blood (BTL, Poland) for total bacteria enumeration. The plated agar media were incubated in gas-tight boxes with anaerobic gas generating sachets (AnaeroGen, Thermo Scientific Oxoid) and incubated at 37 °C for 48 h (MRS agar plates) or 72 h (Columbia agar plates). After incubation, the bacterial colonies were counted and the bacterial cells counts in feces were calculated.

### Statistical Analysis

Statistical analysis was performed using Statistica for Windows 10.0 (StatSoft, Kraków, Poland). The data were expressed as arithmetic means ± standard deviations. The Shapiro–Wilk test was used to check the variables’ normal distribution. Comparison between groups was carried out using one-way ANOVA analysis of variance with Tukey’s post hoc test. A Pearson correlation test was performed to calculate correlation coefficients. A *p* value of less than 0.05 was regarded as significant.

## Results

The masses of the organs removed during the sectioning are presented in Table 1. The liver mass was significantly lower in both supplemented groups than in the control group. The mass of the pancreas was higher in the PB group than in the KK and PA groups.

**Table 1:** Masses of organs

Group	*n*	Liver [g]	Heart [g]	Kidney [g]	Spleen [g]	Pancreas [g]	Femur [g]	Testicle [g]	Duodenum [g]
KK	10	12.136 ± 1.267^b^	1.094 ± 0.055	1.148 ± 0.004	0.575 ± 0.052	0.874 ± 0.095^a^	0.936 ± 0.083	3.578 ± 0.247	0.332 ± 0.050
PA	10	10.456 ± 1.260^a^	1.097 ± 0.082	1.096 ± 0.005	0.556 ± 0.070	0.978 ± 0.141^a^	0.903 ± 0.066	3.629 ± 0.298	0.345 ± 0.065
PB	10	10.361 ± 1.560^a^	1.113 ± 0.102	1.860 ± 0.013	0.517 ± 0.100	1.051 ± 0.126^b^	0.934 ± 0.062	3.535 ± 0.270	0.350 ± 0.059

Data are presented as mean ± SD. *KK*, control group; *PA*, group with low dose of probiotic; *PB*, group with high dose of probiotic; *SD*, standard deviation

^a,b^significantly different (*p* < 0.05)

TIBC and UIBC values and iron serum concentrations are presented in Table 2. At the completion of the experiment, UIBC was significantly higher in the PB group than in the KK group. On the contrary, Fe serum concentration was significantly lower in both PA and PB groups than in the KK group, with no differences between the groups receiving probiotics.

**Table 2:** TIBC and UIBC values and Fe serum concentration

Group	*n*	TIBC [μg/dl]	UIBC [μg/dl]	Fe serum concentration [μg/dl]
KK	10	528.88 ± 35.88	349.28 ± 35.23^a^	171.91 ± 27.68^b^
PA	10	521.40 ± 24.70	388.99 ± 33.72^ab^	132.44 ± 17.86^a^
PB	10	520.22 ± 36.62	395.40 ± 45.29^b^	134.56 ± 7.76^a^

Data are presented as mean ± SD. *KK*, control group; *PA*, group with low dose of probiotic; *PB*, group with high dose of probiotic; *TIBC*, total iron binding capacity; *UIBC*, unsaturated iron-binding capacity; *SD*, standard deviation

^a,b^Significantly different (*p* < 0.05)

The results of whole-blood morphological analysis are presented in Table 3. They reveal that upon completion of the study, the platelet concentration was significantly higher in the PB group than in the KK group.

**Table 3:** Whole blood morphological analysis

Group	n	RBC [T/l]	HGB [g/l]	HCT [l/l]	MCV [f/l]	MCH [pg]	MCHC [g/l]	PLT [g/l]
KK	10	8.58 ± 0.38	147.13 ± 6.64	0.50 ± 0.03	58.13 ± 1.15	17.19 ± 0.36	295.88 ± 5.54	862.71 ± 152.95^a^
PA	10	8.63 ± 0.32	147.50 ± 2.95	0.50 ± 0.01	57.96 ± 1.33	17.12 ± 0.51	295.10 ± 4.58	930.20 ± 149.41^ab^
PB	10	8.88 ± 0.47	152.00 ± 7.60	0.51 ± 0.02	57.69 ± 1.28	17.12 ± 0.36	296.60 ± 3.69	1077.00 ± 169.62^b^

Data are presented as mean ± SD. *KK*, control group; *PA*, group with low dose of probiotic; *PB*, group with high dose of probiotic; *RBC*, red blood cells; *HGB,* hemoglobin serum concentration; *HCT*, hematocrit; *MCV*, mean corpuscular volume; *MCH*, mean corpuscular hemoglobin; *MCHC*, mean corpuscular hemoglobin concentration; *PLT*, platelets; *SD*, standard deviation

^a,b^Significantly different *(p* < 0.05)

The results of the microbiological analysis are presented in Table 4. At the end of the study, the total fecal bacteria content was significantly higher in the PB group than in the PA group.

**Table 4:** Fecal microbiological analysis

Group	*n*	Total fecal bacteria content (T)	*Lactobacillus* fecal content (Lb)	Lb/T	Lb/KK-Lb
KK	10	9.658 ± 0.093^ab^	9.479 ± 0.220	0.981	1.000
PA	10	9.60 ± 0.23^a^	9.52 ± 0.33	0.99	1.004
PB	10	9.931 ± 0.133^b^	9.697 ± 0.117	0.976	1.023

Data are presented as mean ± SD. *KK*, control group; *PA*, group with low dose of probiotic; *PB*, group with high dose of probiotic; bacterial content in feces (T, Lb) given in Log(CFU/g); *KK-Lb*, *Lactobacillus* fecal content in KK group; *SD*, standard deviation

^a,b^Significantly different (*p* < 0.05)

The mineral concentration in tissues is shown in Table 5. Iron content in the liver was significantly higher in the PB group than in the KK group; in the pancreas, this was significantly higher in the PB group than in the KK and PA group, and in the duodenum, it was significantly higher in both supplemented groups than in the KK group. The zinc content in the heart was significantly higher in the PA group than in the KK group; in the testicles, this was significantly higher in the PA and PB groups than in the KK group, and in the pancreas, it was significantly lower in the PA and PB groups than in the KK group. The copper content in the liver was significantly higher in the PA group than in the KK group and in the heart was significantly lower in the PB group than in the KK and PA groups.

**Table 5:** Mineral concentration in tissues

	Group	Liver	Heart	Kidney	Spleen	Pancreas	Femur	Testicles	Duodenum	Hair
Fe [μg/g]	KK	393.19 ± 72.43^a^	605.70 ± 61.31	340.85 ± 52.69	2568.93 ± 357.76	67.00 ± 6.34^a^	168.85 ± 25.83	101.46 ± 16.62	82.83 ± 18.25^a^	12.28 ± 1.65
	PA	438.77 ± 63.38^ab^	657.28 ± 143.48	336.36 ± 22.48	2514.93 ± 222.39	70.65 ± 16.87^a^	164.88 ± 18.04	122.75 ± 23.05	165.06 ± 41.56^b^	11.97 ± 3.42
	PB	479.98 ± 75.38^b^	619.35 ± 82.06	360.22 ± 45.55	2276.99 ± 371.80	79.97 ± 22.26^b^	178.06 ± 23.77	119.95 ± 16.20	166.81 ± 169.61^b^	9.56 ± 1.28
Zn [μg/g]	KK	72.41 ± 7.61	52.97 ± 7.57^a^	69.26 ± 12.25	56.45 ± 11.73	62.43 ± 16.93^b^	981.13 ± 136.52	70.96 ± 26.73^a^	< LOD	124.72 ± 31.84
	PA	84.12 ± 16.22	64.97 ± 10.13^b^	57.82 ± 28.01	63.60 ± 21.31	50.61 ± 11.34^a^	1008.97 ± 220.13	99.07 ± 19.84^b^	< LOD	116.60 ± 12.58
	PB	77.73 ± 14.62	59.77 ± 9.56^ab^	65.94 ± 15.88	62.93 ± 15.10	54.96 ± 21.18^a^	1040.94 ± 269.07	96.47 ± 25.02^b^	< LOD	112.92 ± 9.96
Cu [μg/g]	KK	8.30 ± 1.41^a^	15.45 ± 1.96^b^	19.12 ± 2.97	< LOD	2.61 ± 0.67	6.05 ± 0.33	< LOD	< LOD	< LOD
	PA	10.32 ± 2.23^b^	13.06 ± 2.83^b^	18.93 ± 3.38	< LOD	3.58 ± 0.91	5.56 ± 0.79	< LOD	< LOD	< LOD
	PB	9.82 ± 1.60^ab^	9.70 ± 0.97^a^	19.55 ± 4.06	< LOD	3.50 ± 0.91	5.49 ± 0.83	< LOD	< LOD	< LOD

Data are presented as mean ± SD. *KK*, control group; *PA*, group with low dose of probiotic; *PB*, group with high dose of probiotic; *SD*, standard deviation; *LOD*, limit of detection. Mineral content are presented as Fe, Zn, and Cu content in μg per g of organ dry mass

^a,b^Significantly different (*p* < 0.05)

Correlation analysis of the entire study population (*n* = 30 rats), comparing the determined parameters, was performed: a range of positive correlations were found between the Fe content of the pancreas and UIBC, between the Cu content of the kidneys and RBC count, between the Cu content of the pancreas and UIBC, and between the Fe content of the liver and the Fe content of the pancreas; a negative correlation was seen between PLT count and serum Fe concentration. The significant correlations found in this study are presented in Table 6.

**Table 6:** Significant (*p* < 0.05) correlations registered in the study

Correlated parameters	*r*
Fe pancreas and UIBC	0.52
Cu kidney and RBC	0.53
Cu pancreas and UIBC	0.61
Fe liver and Fe pancreas	0.42
PLT and Fe serum	− 0.50

*RBC*, red blood cells; *PLT*, platelets count; *UIBC*, unsaturated iron-binding capacity

## Discussion

According to the FAO/WHO definition, the essence of probiotics is their viability and beneficial effect on the host after administration in the appropriate amounts [29]. Our study registered significantly higher total fecal bacteria levels in the PB group than in the PA group and higher (though not significant) total fecal bacteria content in the PB group than in the control group. This shows that the supplemented probiotic bacteria remained alive in the rats’ gastrointestinal track and administration in a higher dose resulted in greater intestinal bacterial abundance. It can be hypothesized that the higher of the two probiotic doses has a greater effect on host health. Our study positively reflects this hypothesis, showing more significant differences in analyzed parameters between the PB and KK groups than between the PA and KK groups.

A range of significant differences were also found in the examined parameters between the PA and KK groups, despite there being no significant difference in the total fecal bacteria content between these groups. It can thus be hypothesized that the multispecies probiotic mixture administered at the lower dose has the ability to alter gut microbiota composition, but not its quantity, bringing significant health effects to the host. Aktas and al. have shown that the probiotic *Lactobacillus* in particular*,* which dominates in the probiotic mixture administered here can induce significant alterations in intestinal microbiota composition, altering the abundance of bacteria such as *Bacteroidales, Lachnospiraceae, Oscillospira, Ruminococcaceae, Clostridiales, Clostridia*, and *Firmicutes*. Two main mechanisms lead to these alterations: changes in the regulation of pattern recognition receptor (PRR), which is responsible for cytokine activity modulation in response to bacterial surface patterns, and modifications of antimicrobial peptide (AMP), which is a component of the innate immune system responsible for gut mucosal defense [36]. In our study, the diversity of the hosts’ gut microbiota response to probiotics was shown by the diversity of total fecal bacteria content (demonstrated by its standard deviation), which was higher in the probiotic-supplemented groups than in the control group. However, *Lactobacillus* remained the most abundant bacteria in feces in all three study groups.

Dietary iron absorption is only possible in the form of Fe^2+^ ion and takes place in the duodenum and small intestine. In the intestinal mucosa, iron is bound with apoferritin, creating ferritin, which is then stored in the liver. The iron in ferritin has the form of ion Fe^3+^. In blood, iron is bound to transferin, which is responsible for blood iron transportation [12]. In our study, we found significantly higher duodenal iron levels in both groups that received probiotics, compared to the controls, which indicate higher duodenal iron absorption in these groups. This demonstrates the ability of probiotics to increase iron bioavailability. In vitro studies have shown that probiotic bacteria increase dietary iron bioavailability through a complex mechanism. In the first stage, bacteria converts ellagic acid (EA) to urolithin A (UA) which, unlike EA, is not capable of binding Fe^3+^ [37]. Secondly, Fe^3+^ is reduced to Fe^2+^ by the p-hydroxyphenyllactic acid excreted by Lactobacillus, which increases the amount of the form of Fe that can be absorbed by the host [38]. In our study, *Lactobacillus* fecal content in the PB group was the highest (though not significantly) of all three study groups. This enables us to hypothesize that the above mechanism [37, 38] was crucial in increasing the duodenal iron absorption found in our experiment as a result of multispecies probiotic supplementation. We can emphasize that the probiotic mechanism involving UA, EA, and p-hydroxyphenyllactic acid, which lead to an increase in Fe duodenal absorption, to date, has only been described in in vitro studies; confirmation of its impact on Fe metabolism in living organisms requires further research.

Aside from the increased Fe availability, our study documents elevated liver Fe content as a result of probiotic supplementation in the higher of the two doses, compared to the controls. This finding was accompanied by lower serum Fe concentrations in both supplemented groups compared to the controls; however, there was no anemia, due to hemoglobin values remaining in the normal range and being undifferentiated between groups. We can thus state that probiotic supplementation resulted in a shift of Fe from the blood to the liver and elevated liver Fe accumulation, especially in the group supplemented with a higher dose of probiotic. In adult rats, hematopoiesis takes place in the bone marrow and hematopoietic stem cells (HSC), and nestin^+^ mesenchymal stem cells (MSC) are directly involved in this process [39]. In adult rats, the liver also provides an appropriate environment for migrating HSC. Hepatic stellate cells also present the expression pattern of marrow MSC and exert their function; they are thus liver-resident MSC [39]. When serum Fe concentrations drop, adult rats can (at least partially) shift to liver hematopoiesis [39]. Moreover, a decrease in iron serum content can lead to an increase by as much as a factor of 3 in the levels of hepatic divalent metal-ion transporter-1 protein and divalent metal-ion transporter-1 gene expression, which augments liver Fe uptake [40]. Thus, it can be presumed that probiotic supplementation in multistrain mode may be able to ameliorate hepatic hematopoiesis and hepatic Fe intake. On the other hand, hepatic iron overload might be responsible for increased oxidative stress, nonalcoholic steatohepatitis, and hepatic cancer [41]. Thus, the effect of probiotic supply on the liver needs further investigation, with special concern being paid to the health effects of hepatic iron accumulation. However, it is worth noticing that, in our study, we observed that liver mass in rats supplemented with probiotics was lower than in controls [31]. Elevated liver mass in individuals not consuming alcohol is caused by increased lipid accumulation and oxidative stress [42]. This allows us to hypothesize that multistrain probiotic supplementation may prevent excess lipid storage in the liver [31].

Interestingly, in the PB group, higher platelet counts were observed than in the controls. Moreover, in the entire study population, the platelet count correlated negatively with serum Fe content. Decreased serum iron content is in most cases accompanied by normal platelet count (84.6%), but both thrombocytosis (13.3%) and thrombocytopenia (2.1%) have been observed [43]. Thrombocytosis induced by decreased serum Fe content does not usually lead to clinical consequences. Its mechanisms have not been investigated sufficiently so far. It has been suggested that platelet production might be stimulated by increased erythropoietin. The amino acid sequence homology of erythropoietin and thrombopoietin may also play a role in this process [43].

The study group receiving the higher dose of probiotics was characterized by higher pancreatic Fe content and higher pancreas mass than the group receiving the lower dose of probiotic and controls. Pancreas Fe content has recently been considered an important element in the pathogenesis of diabetes. Pancreas Fe deficiency results in upregulated transcription of arachidonate 15-lipoxygenase (Alox15), a molecule involved in the development of diabetes. On the other hand, increased pancreatic Fe content leads to upregulation of Reg1a, Reg3a, and Reg3b transcription [44]. The Reg genes are a family of islet-derived genes highly expressed in pancreatic stress [44]. It can thus be hypothesized that the increased pancreatic Fe content observed in our study upon supplying probiotics in the higher of the two doses may constitute a biological stress factor in the pancreas. The main mechanism of pancreatic cell damage due to pancreas Fe overload is intensified oxidation [45]. However, Reg genes are also engaged in pancreatic islet regeneration [46] and protection against diabetes [47].

Cu pancreas deficiency results in pancreas atrophy [44] and beta-cell neogenesis [48]. Cu deficiency has been demonstrated to induce pancreatic islet hyperplasia and hepatic metaplasia in the pancreas [49]. Islet hyperplasia is engaged in diabetes pathogenesis [50]. In our study, we observed higher (though not significant) pancreatic Cu content in the probiotics groups than in the controls, accompanied with higher pancreatic mass in the PB group than in the controls, as described above. It can thus be theorized that probiotic supplementation in multistrain mode may play a role in the prevention of diabetes and counteract pancreas degeneration.

Interestingly, correlation analysis of the entire study population revealed that Fe content in the liver and pancreas was positively correlated. This is due to common Fe transporters in both the liver and pancreas: ZRT/IRT-like protein 14 (ZIP14) and divalent metal-ion transporter-1 (DMT1) [40], which is also engaged in Cu metabolism [51]. Moreover, the ZIP14 level is upregulated in the Fe-loaded pancreas and liver [40]. In the entire study population, we registered a positive correlation between the UIBC level and the pancreas Fe content. Elevated UIBC level is a marker of a decrease in serum Fe concentration [52]. This observation of a reduction in iron serum content proportional to an increase in iron pancreas content confirms our hypothesis on the shift of iron from the serum to parenchymal organs, such as the liver and pancreas, as a result of multistrain probiotic supplementation. In our study, UIBC level was also positively correlated with copper pancreas content in the entire study group. These data suggest that the observed alterations in Cu status may follow on from alterations in Fe homeostasis.

Fe metabolism is strongly connected with Cu and Zn homeostasis, thus studies of Fe alterations should also include Cu and Zn content analysis [22]. Studies on the effects of probiotic supply on Cu and Zn status are very scant. Despite this, it has been shown that probiotic supplementation in rats may correct such mineral imbalance [53]. In our study, we observed higher liver Cu contents in the group receiving the lower dose of probiotics than in the controls, accompanied by lower heart Cu content in the PB group than in the KK and PA groups. It can thus be hypothesized that the multistrain probiotic supply has led to a shift in Cu ions. The liver Cu deficiency plays a role in the pathogenesis of diseases such as nonalcoholic fatty liver disease, bile duct ligation-induced liver injury, fibrosis, inhibited ceruloplasmin activity, and disturbed heme oxygenase-1 gene expression [54–56]. Multistrain probiotic supplementation that increases hepatic Cu content may thus play a protective role against a range of liver diseases. On the other hand, heart Cu deficiency may lead to unfavorable copper-deficient heart hypertrophy [57]. However, the observed shift of Cu ions is probably secondary to the changes in Fe status [22]. There is an evident need for further studies on Cu status alterations as the result of probiotic supplementation. In the entire study population, we observed a positive correlation between kidney Cu content and red blood cell (RBC) count. The probiotic-supplemented rats did not show markers of anemization, compared to the controls, despite the lower Fe serum content. We thus hypothesize that renal Cu content takes part in preventing anemia though a mechanism that has so far not been well investigated. It is worth underlining that probiotic supplemented rats showed no differences in renal Cu levels when compared to the controls [58].

Our study showed lower zinc content in the pancreas in groups receiving probiotic than in the control group, accompanied by the above-described higher levels of pancreatic Fe in the group receiving higher doses of probiotic than the controls and the group receiving the lower dose of probiotic. Zn plays a protective role in the pancreas against the unfavorable effects of oxidative stress [59]. A decrease in pancreatic Zn content, and also the oxidative stress caused by an increase in pancreas Fe content [45], may cause damage to pancreatic tissue [59]. Moreover, zinc is an element crucial to undisturbed insulin synthesis, and a deficit of it may result in disturbances to glucose metabolism [60]. We thus hypothesize that multistrain probiotic supplementation, on account of alteration to pancreatic Zn and Fe contents, may increase the risk of the development of diabetes. This issue undoubtedly needs further investigation. However, the pancreas has mechanisms to minimize carbohydrate metabolism disorders, despite zinc deficiency [60].

Zn plays a protective role against homocysteine-induced peroxidation-based cardiac disorders [61], and its deficiency leads to unfavorable enzymatic alterations in the heart [62]. In our study, we found heart Zn contents to be higher in the PA group than in the KK group. It can thus be hypothesized that multistrain probiotic supplementation can serve as an intervention to prevent potential Zn-deficiency-derived heart disturbances. Moreover, it allows us to suppose that the Zn-dependent cardioprotective effect of probiotic supply would counteract the development of copper-deficient heart hypertrophy [57], which may be an effect of lower heart Cu content in the PB group than in the KK and PA groups in our study after probiotic supply. This issue requires deeper research efforts.

There is an evident dearth of studies on Zn metabolism in the testicles. In one study, Vanderlei et al. demonstrated that zinc deficiency leads to germinative epithelium degeneration, atrophy of seminiferous tubules, and spermatogenesis disturbances [63]. Our study found higher testicle Zn content in both groups receiving the probiotic than in the control group, which allows us to theorize that a multistrain probiotic supply would prevent the testicular disturbances observed by Vanderlei et al.

### Study Strengths

The strongest point of our study is its pioneering character, emplying multistrain probiotic supplementation and a dose-comparatory mode not previously implemented in studies of the effect of probiotic supplementation on Fe, Zn, and Cu status. Our study can serve as a basis for future worldwide guidelines on probiotic supplementation.

### Study Limitations

The main limitation of our study was its slightly limited duration. However, an experiment as short as 6 weeks was sufficient to detect significant and partially dose-dependent effects of multistrain probiotic supply on the selected parameters of iron status. Moreover, our study was performed only on healthy males, omitting females and rats in a state of illness.

## Conclusion

Our study has provided evidence for dose-independent and beneficial effect of 6-week oral multispecies probiotic supplementation on iron bioavailability and duodenal iron absorption in the rat model. Moreover, multistrain probiotic supply induces a dose-independent iron shift from serum and intensifies dose-dependent pancreatic and liver iron uptake, as an effect of as little as 1 × 10^10^ CFU daily dose. The iron status modifications resulting from multispecies probiotic supply were accompanied by a range of changes in copper and zinc status, especially in the heart, pancreas, and testicles; these need further investigation. Subsequent studies on a large scale should be undertaken to draw a precise conclusion, with special attention on the health effects of these mineral alterations.
